# Imatinib, a New Adjuvant Medical Treatment for Multifocal Villonodular Synovitis Associated to Noonan Syndrome: A Case Report and Literature Review

**DOI:** 10.3389/fmed.2021.817873

**Published:** 2022-01-17

**Authors:** Romain Dalla-Torre, Vincent Crenn, Pierre Menu, Bertrand Isidor, Pascale Guillot, Benoit Le Goff, Loic Geffroy, Marc Dauty, Alban Fouasson-Chailloux

**Affiliations:** ^1^CHU Nantes, Service de Médecine Physique et de Réadaptation Locomotrice, University Hospital of Nantes, Nantes, France; ^2^CHU Nantes, Service de Rhumatologie, University Hospital of Nantes, Nantes, France; ^3^CHU de Nantes, Clinique Chirurgicale Orthopédique et Traumatologique, Hôtel-Dieu, Nantes, France; ^4^CHU Nantes, Service de Médecine du Sport, University Hospital of Nantes, Nantes, France; ^5^INSERM UMR U1229/RMeS, Regenerative Medicine and Skeleton – Nantes University, Nantes, France; ^6^IRMS, Institut Régional de Médecine du Sport, Hôpital Saint Jacques, Nantes, France; ^7^CHU Nantes, Service de Génétique Médicale, University Hospital of Nantes, Nantes, France; ^8^Chirurgie Orthopédique, Santé Atlantique, Saint Herblain, France

**Keywords:** Noonan syndrome, multifocal villonodular synovitis, Imatinib, joints, case report

## Abstract

Noonan syndrome (NS) is an autosomal dominant multisystem disorder caused by the dysregulation of the Rat Sarcoma/Mitogen-activated protein kinase (RAS/MAPK) pathway and characterized by short stature, heart defects, pectus excavatum, webbed neck, learning disabilities, cryptorchidism, and facial dysmorphia. Villonodular synovitis is a joint disorder most common in young adults characterized by an abnormal proliferation of the synovial membrane. Multifocal Villonodular synovitis is a rare disease whose recurrent nature can make its management particularly difficult. Currently, there is no systemic therapy recommended in diffuse and recurrent forms, especially because of the fear of long-term side effects in patients, who are usually young. Yet, tyrosine kinase inhibitors seem promising to reduce the effects of an aberrant colony stimulating factor-1 (CSF-1) production at the origin of the synovial nodule proliferation. We present here the case of a 21-year-old woman with NS associated to diffuse multifocal villonodular synovitis (DMVS). Our clinical case provides therapeutic experience in this very rare association. Indeed, in association with surgery, the patient improved considerably: she had complete daily life autonomy, knee joint amplitudes of 100° in flexion and 0° in extension and was able to walk for 10 min without any technical assistance. To our knowledge, this is the first case of a patient suffering from DMVS associated with a Noonan syndrome treated with Glivec^®^ (oral administration at a dosage of 340 mg/m^2^ in children, until disease regression) on a long-term basis.

## Introduction

Pigmented villonodular synovitis is a benign condition that can affect joints or tendon sheaths. It is characterized by the proliferation of the synovial tissue associated to a deposit of hemosiderin. It can be locally aggressive, leading to joint destruction due to repeated haemarthrosis (1). Chronic swelling and joint pain suggest the clinical diagnosis. MRI is the imaging modality of choice to explore the joint extension of this disease. Histopathology of synovial tissue is recommended in case of doubt to make the diagnosis. This is a rare disease whose recurrent nature can make therapeutic management particularly difficult. Synovectomy is the treatment of choice, under arthroscopy or open surgery depending on the case. However, the recurrence rate remains high (25–50%). Adjuvant techniques such as synoviorthesis and external radiotherapy have proven to be effective but do not prevent long-term recurrence. Currently there is no approved systemic therapy (2).

Noonan syndrome is an autosomal dominant multisystemic disorder subject to genetic heterogeneity. It is characterized by short stature, heart defects, pectus excavatum, webbed neck, cryptorchidism and facial dysmorphia, associated with learning difficulties (3, 4). This developmental disorder is linked to mutations in genes (PTPN11, SOS1, RAF1…) coding for various components of the Rat Sarcoma/Mitogen-activated protein kinase (RAS/MAPK) cell signaling pathway. This signaling cascade is also involved in many other inherited disorders such as cardio-facio-cutaneous syndrome (craniofacial dysmorphia, congenital heart disease, dermatoses, neurological manifestations, growth retardation, and intellectual disability), LEOPARD syndrome (ECG conduction abnormalities, ocular hypertelorism, pulmonary stenosis, abnormal genitalia, retardations of growth, and deafness), Costello syndrome (growth retardation, short stature, developmental delay or intellectual deficit, joint laxity, loose skin and facial dysmorphia) and neurofibromatosis type 1 (café-au-lait spots, Lish nodules, lentigos on the armpits and inguinal region, and multiple neurofibromas) (5). It seems that joint manifestations are frequent, predominant in large joints (6). Despite potentially serious functional consequences, joint involvement has rarely been studied in the literature. Exceptionally, these syndromes may be associated with the development of single or multiple giant cell lesions such as pigmented villonodular synovitis (7). Many genes have been implicated but no geno-phenotype link has been proven so far. Deregulation of the RAS/MAPK cell signaling cascade is thought to be the basic molecular event pre-disposing to the formation of giant cell lesions without direct link to a specific genomic mutation.

We report a clinical case of a rare association, between a Noonan syndrome and a diffuse multifocal villonodular syndrome (DMVS), to share our therapeutic experience.

## Case Presentation

A 21-year-old woman with Noonan syndrome due to a maternally inherited mutation (c.598 A4T, located in exon 5 of the PTPN11 gene) had for several years been suffering from a DMVS, difficult to treat. The patient had characteristic polymorphism (depressed nasal wing, hypertelorism, sloping palpebral slits, posteriorly angled ears with thick helices), stature and weight retardation (Height: 151 cm; weight: 34 kg; BMI: 14.9 kg/m^2^).

In early childhood, the patient presented with a lesion of the right mandibular ramus with a diagnosis of giant cell granuloma. At the age of 7, characteristic MRI images—synovial hypertrophy, joint effusion, bone erosions, enhancement after gadolinium injection and especially hemosiderin deposits in the synovial masses appearing as a low signal area best seen on FFE sequence—strongly suggestive of the diagnosis of multifocal villonodular synovitis were identified in the knees and tibio-tarsal joints. This diagnosis was later confirmed by histopathological examination.

**Figure 1:**
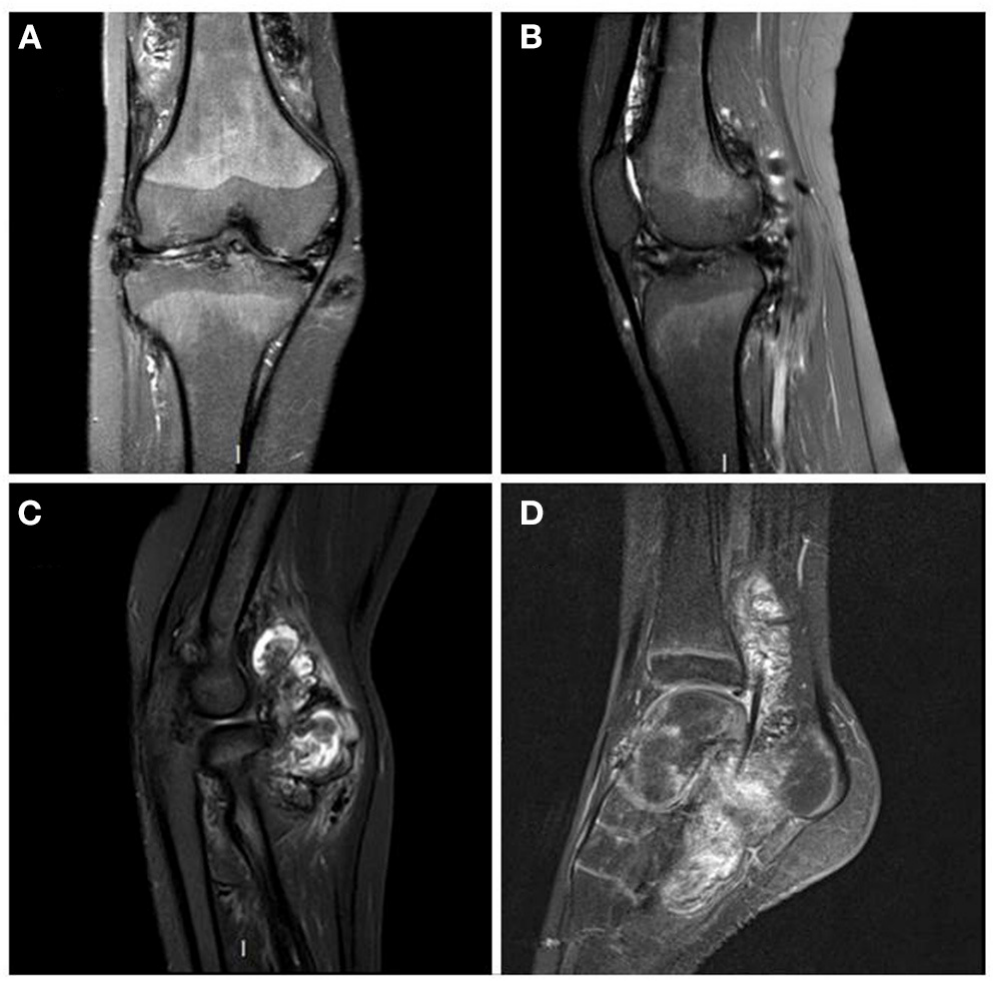
MRI T1 Fat Sat sequences - right knee in coronal plane **(A)** and right knee in sagittal plane **(B)**: diffuse synovial pannus, especially in the superior recess of the joint. Tri-compartmental chondropathy associating stage IV chondrolysis associated with multiple subchondral cysts; STIR sequence of the right elbow in sagittal plane **(C)**: anterior intra-articular synovial masses in heterogeneous signal; T1 sequence after gadolinium injection of left ankle in sagittal plane **(D)**: Large synovial panuses mainly affecting the subtalar joint and voluminous synovial masses developed around the long plantar flexor hallucis behind the lower end of the tibia. These signs were highly suggestive of multifocal villonodular synovitis which was later confirmed by histopathological examination.

This DMVS was then managed by surgical synovectomies with a double approach on each of the four joints (Figure 1). Each surgical synovectomy was completed with Triamcinolone Hexacetonide synoviorthesis in the weeks following the surgical procedure. Rapidly, the first recurrences appeared in each of these joints, and lesions in the wrists appeared at the age of 11. The functional disability was relatively important with walking limited to short trips; the patient moving most of the time in a wheelchair. The evolution of the lesions, particularly in the right knee, led to the introduction of a basic treatment with Methotrexate. This treatment caused asthenia and was ineffective since it did not prevent the reappearance of knee pain associated with a new localization of the disease on the right elbow.

This failure of the initial management motivated a third line of treatment with a tyrosine kinase inhibitor of the Imatinib-type (Sandoz-France) (Glivec^®^), at a dose of 340 mg/m^2^ from the age of 12. This new treatment was rapidly effective and well-tolerated. However, joint pain recurred 6 months after the treatment had been stopped, so it was restarted and prescribed for 3 years. The only notable side effects were intermittent asthenia and nausea, which were corrected by systematically taking an antiemetic before taking Glivec^®^. Unfortunately, the villonodular synovitis progressively recurred in the right knee despite the maximum dose of treatment prescribed. This recurrence was, at least partially attributable to a non-strict treatment compliance.

**Figure 2:**
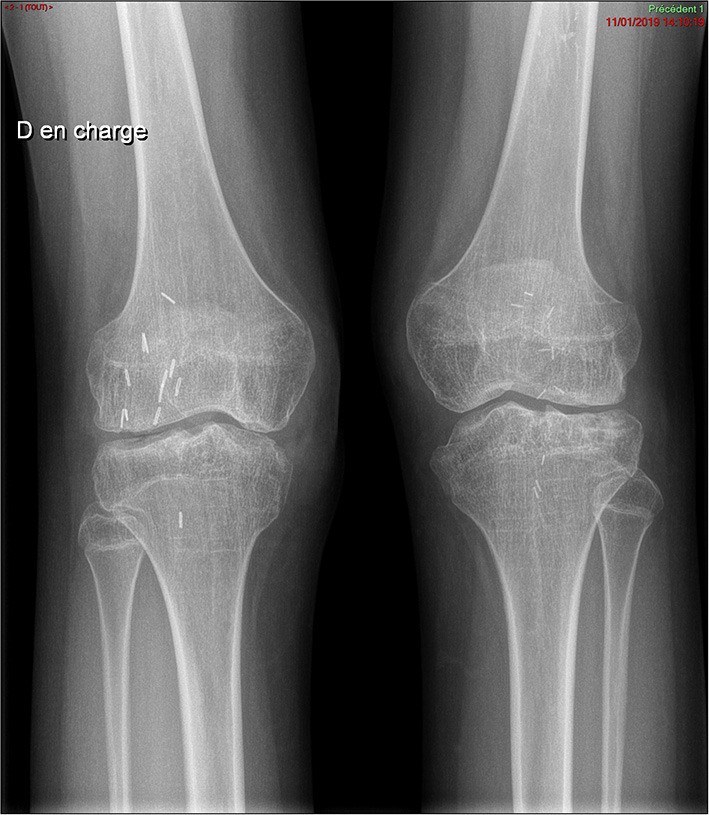
Plain x-ray of the knees. Pancompartmental joint space loss reflecting secondary osteoarthritis changes, with presence of diffuse joint narrowing and subchondral cysts.

Finally, a surgical removal of the synovium allowed an improvement of the pain but required a joint mobilization under general anesthesia because of difficulties in recovering knee flexion. Currently, the patient has recovered complete autonomy, joint amplitudes are at 100° in flexion and 0° in extension, and walking can be performed for 10 min without technical aids. However, the radiographs show tricompartmental space loss, so that total knee prosthesis will probably be considered in the future (Figure 2).

## Discussion

The gold standard for the treatment of pigmented villonodular synovitis is surgical excision by a total synovectomy of the affected joint, using an open or an arthroscopic approach (8). However, the recurrence rate after surgery can be as high as 50%, leaving the possibility of using other treatment options (2). External beam radiation therapy at 30–50 Gy, used as monotherapy, has demonstrated local control of up to 95% while significantly decreasing DMVS recurrence when combined with surgical synovectomy (9).

In giant cell tumors, including villonodular synovitis, a neoplastic clone overexpressing colony stimulating factor-1 (CSF-1) has been described. A t (1;2) translocation that links the CSF-1 gene on chromosome 1p13 to the COL6A3 gene on chromosome 2q35 has been described and is thought to be responsible for the overproduction of CSF-1 by neoplastic cells (10, 11). Inhibition of CSF-1/CSF-1 receptor (CSF-1R) signaling is the target of new systemic therapies such as monoclonal antibodies and tyrosine kinase inhibitors. Among these agents, Imatinib, a tyrosine kinase inhibitor, has been studied in a multicenter retrospective cohort study of patients with recurrent diffuse villonodular synovitis (12). The treatment showed significant improvement (31% of overall response) but numerous adverse events were reported. Indeed, more than half of the patients had discontinued treatment due to drug toxicity.

The development of more specific tyrosine kinase inhibitors including Emactuzumab (SynOx Therapeutics- Ireland) and PLX3397 may have a promising future (2). Emactuzumab is a monoclonal antibody that binds directly to the CSF-1 receptor on the surface of macrophages to reduce or eliminate the effects of aberrant CSF-1 production. In a recent trial, 26 out of 28 patients with DMVS showed significant symptomatic improvement within 1–2 weeks of treatment with Emactuzumab (2). More recently in 2019, the CSF-1 receptor antagonist Pexidartinib (Daiichi Sankyo - France) was approved by the FDA for its use in patients with DMVS who are unlikely to benefit from surgery. There are encouraging results, but they have to be qualified by the persistence of non-negligible side effects, particularly hepatic ones (13).

Initially, the association between Noonan's syndrome and giant cell tumors was considered a separate syndrome (7, 14). It is now accepted that giant cell tumors belong to the spectrum of Noonan syndrome (10), as shown by our patient, who has a mutation transmitted by her mother who has a Noonan syndrome without DMVS.

A recent multicenter retrospective study evaluated the prevalence of joint pain in 71 patients with Noonan syndrome from 4 pediatric centers. The authors concluded that joint manifestations were common in Noonan syndrome, predominated in large joints, and that multiple villonodular synovitis were characteristic but rare (6).

In the literature, there are only few reported cases of association between Noonan syndrome and villonodular synovitis, and none of them reporting a management with Glivec^®^ on long-term follow-up (Table 1).

**Table 1:** Association of Noonan syndrome and villonodular synovitis in the literature.

**References**	**Number of cases**	**Sex**	**Age (years)**	**Unifocal/multifocal**	**Localization**	**Treatment**	**Evolution**
Cohen et al. (7)	2	NA	NA	Multifocal	NA	NA	NA
	1	F	9	Multifocal	Wrists, knees, ankles	Surgery and irradiation	NA
	1	F	7	Multifocal	1 Knee, 2 elbows	Surgery	Recurrence after 6 months
	1	M	4	Unifocal	Knee	NA	NA
	1	M	5	Unifocal	Knee	NA	NA
	1	M	10	Unifocal	Knee	NA	NA
Mascheroni et al. (11)	1	M	13	Unifocal	Ankle	Surgery	NA
Vavrik et al. (15)	1	M	30	Multifocal	Knees	Surgery, irradiation, total knee prosthesis	Multiple recurrences, improvement after the 2 prostheses
Beneteau et al. (14)	1	M	12	Unifocal	Ankle	NA	NA
Meyers et al. (16)	1	M	6	Multifocal	Knees, ankles	Surgery	NA
Miri et al. (17)	1	M	8	Unifocal	Left knee, both elbow	Surgery	NA

*NA, Not available*.

Our clinical case provides therapeutic experience in this very rare association. There are data in the literature regarding the use of Glivec^®^ in the treatment of giant cell tumors including uni and multifocal villonodular synovitis. One of the obstacles to the use of this long-term treatment in young patients is the lack of data on long-term follow-up. To our knowledge, this would be the first case of a patient suffering from DVMS associated with a Noonan syndrome treated with Glivec^®^ on such a long term basis. Few cases of villonodullar synovitis treated with Glivec^®^ have been reported (18–20). Case series show a symptomatic efficacy of around 30% and stabilize the lesions in 60–65% of the cases, whereas discontinuation of treatment due to side effects is encountered in ~12–20% of the cases (Table 2).

**Table 2:** Clinical experiences with Imatinib.

**References**	**Design**	**Type of TGCT**	**Number of patients on Imatinib**	**Duration of treatment (median)**	**Efficiency on symptoms**	**Stability of the disease**	**Tolerance**
Cassier et al. (21)	Clinical series	NA	29	4–7 months	74%	73%	22% of Toxicity
Verspoor et al. (12)	Retrospective study	NA	62	9 months	31%	65%	12% stopped treatment for toxicity AE grade 1–2: 78% AE grade 3: 9%
Mastboom et al. (22)	Retrospective study	NA	25	7 months	32%	63%	12% stopped treatment for toxicity AE grade 1–2 : 80% AE grade 3 : 12%
Brahmi et al. (23)	Retrospective study	NA	15	6 months	1st line of treatment:11%	60%	3 patients stopped treatment because of AE
					2nd line: 6%	14%	
					3rd line: 3%	66%	

*NA, not available; TGCT, Tenosynovial Giant Cell Tumors; AE, adverse effects*.

In case of failure or non-tolerance, surgery makes it possible to reduce the tumor inoculum as much as possible in order to recover indolence and joint mobility thanks to rehabilitation care. Thus, the handicap of movement is reduced when a joint such as the knee or the ankle is treated. It is hoped to improve the quality of life while delaying the destruction of the joint, which will eventually require the replacement of the joint with a prosthesis. Indeed, DMVS is a potentially aggressive benign tumor which, as in our patient, is difficult to control. A multidisciplinary approach with surgeons, rheumatologists, radiologists, oncologists and rehabilitation physicians is essential.

## Conclusion

Multifocal villonodular synovitis is rarely associated to Noonan syndrome due to a link with a deregulation of the RAS/MAPK cell signaling cascade responsible for giant cell tumor formation. Treatment is multidisciplinary with priority given to surgical synovectomy. Adjuvant treatments exist for diffuse recurrent forms such as external radiotherapy or synoviorthesis. No systemic therapy is currently recommended, especially because of the fear of long-term side effects in patients who are usually young, but, if the side effects are tolerated, tyrosine kinase inhibitors seem promising to reduce the effects of aberrant CSF-1 production at the origin of synovial nodule formation.

## Data Availability Statement

The raw data supporting the conclusions of this article will be made available by the authors, without undue reservation.

## Ethics Statement

Written informed consent was obtained from the patient for publication of this case report.

## Author Contributions

RD-T, MD, and AF-C: conceptualization, methodology, and writing—original draft preparation. MD and PM: formal analysis. LG, VC, PG, BL, and BI: surgery and patient management. PM, LG, VC, PG, BL, and BI: writing—review and editing. All authors have read and agreed to the submitted version of the manuscript.

## Conflict of Interest

The authors declare that the research was conducted in the absence of any commercial or financial relationships that could be construed as a potential conflict of interest.

## Publisher's Note

All claims expressed in this article are solely those of the authors and do not necessarily represent those of their affiliated organizations, or those of the publisher, the editors and the reviewers. Any product that may be evaluated in this article, or claim that may be made by its manufacturer, is not guaranteed or endorsed by the publisher.
